# High-resolution analysis of ordered and disordered isoporous 3D nanostructures using PXCT

**DOI:** 10.1186/s11671-026-04435-7

**Published:** 2026-02-17

**Authors:** Birte Hindenlang, Antoine E. Jimenez, Tobias Krekeler, Martin Ritter, Ana Diaz, Mirko Holler, Yen Häntsch, Kaline P. Furlan, Berit Zeller-Plumhoff

**Affiliations:** 1 Institute of Metallic Biomaterials, Helmholtz-Zentrum Hereon, Geesthacht, Germany; 2 Karlsruhe Institute of Technology (KIT), Institute for Applied Materials – Ceramic Materials and Technology, Karlsruhe, Germany; 3 Electron Microscopy Unit, Hamburg University of Technology, Hamburg, Germany; 4 Paul Scherrer Institute, PSI Center for Photon Science, Forschungsstrasse 111, Villigen PSI, 5232 Switzerland; 5 Institute of Advanced Ceramics, Research conducted while at Hamburg University of Technology, Hamburg, Germany; 6 Faculty of Mechanical Engineering and Marine Technologies, University of Rostock, Data- Driven Analysis and Design of Materials, Rostock, Germany

## Abstract

Many applications of 3D isoporous (nano)structures rely on the use of liquids flowing through the material. Therefore, a better understanding of the underlying processes and the influence of the structural properties on the fluid flow is of high importance. However, due to the small structure sizes, the analysis of the fluid flow processes is difficult. Using synchrotron radiation-based ptychography X-ray computed tomography (PXCT), ordered and disordered isoporous Al_2_O_3_ structures with different nominal pore sizes are investigated and characterized. The detailed, scanned 3D structures are simulated regarding their differences in fluid flow properties. It was found that ordering has only a minor influence on the fluid velocity’s homogeneity compared to the influence of the connection points between the macropores. Keywords: Ptychography X-ray CT, permeability, isoporous nanostructures, fluid flow simulation.

**Supplementary Information:**

The online version contains supplementary material available at 10.1186/s11671-026-04435-7.

## Introduction

 Many processes in nature, as well as in modern technology, rely on functional porous materials [1, 2], for example, catalysis, sensors, biomedicine, energy conversion, or storage [3, 4]. In the field of photonics, 3D isoporous photonic crystals and glasses, also named inverse opals, and surface-templated photonic glasses, are used for tailored reflection of light, enabling applications such as structural colors [5] or broadband unidirectional reflectors [6]. By varying the pore diameter, different wavelengths are affected. The structures considered in this work have macropore diameters of 900 nm and1500 nm. Therefore, they are responsive in the near infrared regime. In many of the applications, the performance of these materials is primarily governed by the properties of their interfaces, including surface morphology and their interactions with the surrounding environment.

Generally, 3D isoporous structures based on inverse opals or photonic glasses are highly porous structures with a high degree of interconnected pores due to the template-based fabrication method [7]. In combination with small, sub-micrometer pore sizes, the open porosity enables a high permeability and large surface-to-volume ratio, which is ideal for heat and mass transport [8]. These properties can be further altered by chemically functionalizing the structure’s surface to enhance or hinder infiltration of the pore network [9, 10]. Moreover, the fraction of porosity and the pores’ properties can be altered to further tune their fluid transport characteristics [11, 12]. Additionally, by changing the pore size of the porous structure, its permeability can be further altered. Exemplarily, the pores’ volumetric distribution in the 3D structure can be designed to be either ordered or disordered, which may lead to differences in the tortuosity of any fluid flow path and, thus, the fluid permeability of the structure [8]. As is well known, the connection point or pore throat size has a decisive impact on the permeability and, therefore, on the fluid behavior in porous structures [12–15]. It is now of interest how a change in ordering affects the fluid behavior.

Overall, the interaction of 3D (nano)structures with fluids, such as water, can enable further functionalities, including switchability in terms of their properties. As an example, by infiltrating the porous structure with water, band gaps and reflective indices can be tuned, opening new optical applications [10]. However, in this case, the infiltration must be sufficient to achieve the required accuracy in optical property tuning. Therefore, it is necessary to understand the fluid flow inside different porous structures. Furthermore, when the pores are in the sub micrometer to nanometer range, fluid confinement effects may arise. This is due to the small length scale of the fluid transport paths and the interaction of the fluid with the materials’ interfaces [16]. Therefore, investigating the fluid transport processes at the nano to submicron scale is challenging for reduced pore sizes, and the interaction between the nanoporous material and fluid is still not fully understood.

Using conventional 2D analysis or 2D views, only the surface or one material layer can be characterized per view. However, to understand the fluid flow through the 3D nanostructures, a 3D visualization is needed. Several methods are possible to achieve a 3D visualization of the inner porous structure. However, methods like electron tomography require samples in the nanometer to low micrometer range. To adequately investigate and simulate the fluid flow in porous structures with pore sizes above 900 nm, these sample dimensions are not sufficient. A non-destructive technique allowing for larger sample sizes is X-ray computed tomography (CT). It has been demonstrated to characterize the inner structure and morphology of porous materials [17]. However, in the case of nanomaterials, CT at laboratory sources does not provide sufficient resolution to resolve the intricate details of the samples. Synchrotron radiation-based techniques have the potential to provide better spatial resolution, owing to the high brilliance of synchrotron sources [18, 19]. In particular, ptychographic X-ray computed tomography (PXCT) enables high-resolution imaging of structural features ranging from the micrometer to the nanometer scale. It has been demonstrated to obtain high-resolution 3D reconstructions of isoporous samples [5, 7, 20]. Compared to other synchrotron radiation-based methods, PXCT offers state-of-the-art resolutions with lower noise levels.

In the present study, both ordered and disordered 3D isoporous Al_2_O_3_ samples with pore sizes in the submicrometer range are investigated by PXCT, and their fluid transport behavior is compared using image-based modelling. Based on the images, the samples are characterized regarding their surface area, volume fraction, and size distribution of the pore spaces. The 3D reconstructions obtained from synchrotron PXCT experiments are further used to simulate the fluid permeability of the different structures and to correlate them with their structural properties. This simulation provides a first understanding of the influence of the structural parameters on the fluid behavior. The results show an intricate correlation between the permeability, the macropore size, the tortuosity, and the connection point sizes. These new insights provide input for the future fabrication of such isoporous 3D (nano)structures with tailored fluid transport behavior.

## Materials and methods

### Material preparation

**Fig. 1:**
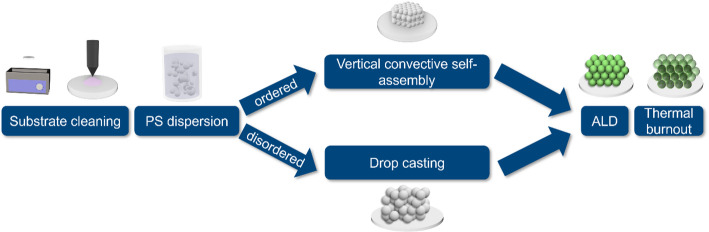
Schematic workflow of the material preparation. In the first step, the substrate is cleaned. Afterwards, the polystyrene (PS) dispersion is deposited on the substrate in an ordered or disordered assembly. By atomic layer deposition (ALD), the Al_2_O_3_ is deposited, followed by thermal burnout to remove the PS

The ceramic-based isoporous 3D structures were fabricated by a template-based method consisting of self-assembly of polymeric templates onto sapphire substrates ($\left\langle {10\bar{2}0} \right\rangle$ 25 × 20 mm, Crystec GmbH), followed by atomic layer deposition (ALD) and template removal (see Fig. 1). Prior to the self-assembly process, the sapphire substrates were ultrasonically cleaned for 1 h in a 1 wt% detergent (Mucasol Brand, Merz Hygiene GmbH) ultrapure water (miliQ H_2_O) solution, followed by brushing, rinsing, drying with nitrogen, and oxygen-plasma treated for 20 min (Polaron PT7160, Quorum Technologies). Dispersions of monodisperse polystyrene particles (Microparticles GmbH) with diameters of either 0.9–1.5 μm at a concentration of 1 mg ml^− 1^ and 15 mg ml^− 1^ were used for the fabrication of ordered and disordered structures, respectively. For ordered structures, a vertical convective self-assembly process was performed within a humidity chamber (Memmert HCP 108) with controlled humidity and temperature of 70% RH and 55 °C for a maximum of 93.5 h. For the disordered structures, drop casting of 160 µL dispersion was performed onto substrates pre-heated for 15 min at 60 °C. To avoid the assembly into an ordered structure, homocoagulation was induced by adding 1 mol/L HCl solution as described in one of our previous works [21]. In the following, we will refer to the four different samples by stating the diameter of the particles used to manufacture them and their state of order. It must be noted that the resulting pore sizes differ from the initial particle sizes, and the named diameter is just the mean diameter of the pores.

After self-assembly, a low-temperature ALD process of Al_2_O_3_ was carried out at 95 °C in a home-made reactor under exposure modus using nitrogen as carrier gas. Trimethylaluminum (TMA min. 98%, Strem chemicals) and miliQ H_2_O were used as precursors to generate aluminum oxide coatings onto the 3D templates. The precursors’ pulse, pump, and exposure time were 0.1/20/90 and 0.2/20/90 seconds, respectively. After ALD, the template was removed via thermal burnout in a muffle furnace under an air atmosphere with a ramp up of 0.3 °C min-1 up to 500 °C, where the samples were kept for 30 min. Therefore, producing highly porous isoporous structures, either ordered or disordered structures, with two different pore sizes, corresponding to the two different template particle diameters (0.9 and 1.5 μm). Before preparing pillars for the tomography measurements, the resulting samples were analyzed by scanning electron microscopy (SEM, Zeiss Supra 55 VP).

Focused ion beam (FIB, FEI Helios G3 UC) milling was employed to fabricate cylindrical pillars of ~ 12 μm diameter from the isoporous 3D structures, which were pre-coated with carbon using a carbon thread evaporator (Bal Tec CED 030) to ensure conductivity across the sample. The pillars were created by a circular milling pattern with a beam current of 9.3 nA. An in-situ nanomanipulator was employed at a 0° sample tilt to transfer the pillar to the tomography pins, with platinum ion deposition securing the transfer. A cleaning cross-sectioning pattern with a 0.43 nA beam current was then used to remove the platinum and any residual tungsten from the manipulator needle at the top of the sample. The final polishing step was completed using an 80 pA beam current.

### Ptychographic X-ray computed tomography (PXCT) data acquisition and processing

**Fig. 2:**
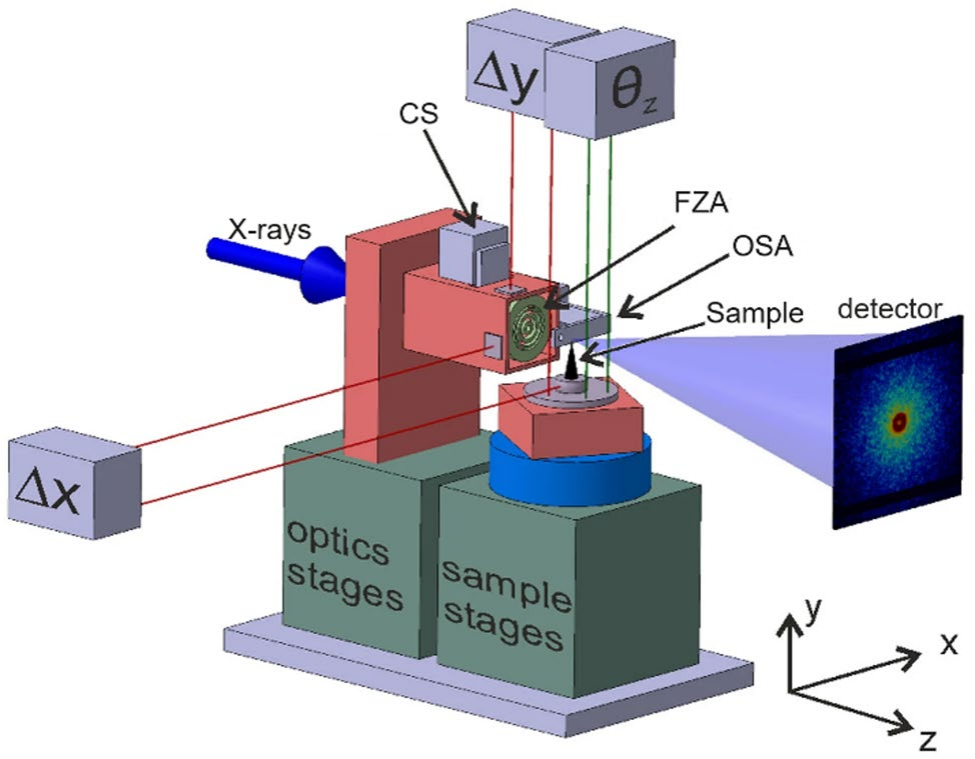
Schematic overview of the beamline setup. Indicated are the X-ray direction, the central stop (CS), the Fresnel zone plate (FZA), the order sorting aperture (OSA), the sample, and the detector. (adapted from [22]).

The FIB-prepared pillars were mounted onto OMNY pins designed specifically for the ptychographic X-ray computed tomography (PXCT) measurements. These were conducted at the cSAXS beamline of the Swiss Light Source (SLS) at the Paul Scherrer Institute (PSI) in Villigen, Switzerland with a high-resolution PXCT instrument optimized by Holler et al. [22] (see Fig. 2). This instrument integrates relative interferometry between the sample and a Fresnel zone plate that defines the illumination to ensure a relative positioning precision on the order of 5 nm, which is essential for achieving high-resolution PXCT. Measurements were performed at a photon energy of 6.2 keV. In the measurement, the sample is translated through the illumination field with an automated setup, while diffraction patterns are captured on a 2D detector in the far field at each scan position. Iterative phase retrieval algorithms are then employed to reconstruct 2D images, which provide a projection of the refractive index of the sample, displaying both amplitude and phase contrast. Subsequently, the phase images from all 2D projections are tomographically reconstructed to generate a 3D map of the electron density of the sample [23]. Ptychographic reconstructions were carried out using the PtychoShelves software package, developed by the Coherent X-ray Scattering group at the PSI [24]. More details about the PXCT data acquisition and processing can be found in the supplementary information, including an explanation for the choice of critical parameters.

### PXCT analysis

The obtained PXCT reconstructed slices have only minor noise. To ease the further segmentation, a median filter with *r* = 2 px is applied in FIJI [25, 26]. In the following, the filtered data is segmented using the 2D machine learning-based WEKA module available in FIJI [27]. It was trained on 10% of the image stack using the module’s default settings, resulting in a low error.

For each material type, the properties such as surface area and volume fraction are calculated, the macropores and connection points are defined, and the permeability is simulated. All analysis steps are performed in Avizo (Avizo 2021.1, FEI SAS, Thermo Scientific, France). The original segmentation is used to calculate the surface area and the volume fraction of the material. Additionally, the tortuosity $\tau\:$ of the different material types through the original segmentation, meaning the isoporous structure, is calculated by creating a skeletonization. It is calculated with ${d}_{curved}=\sum\:{d}_{points}$, where ${d}_{points}\:$is the Euclidean distance between points of the same curve, and$\:{d}_{straight}$ the direct distance between start and end point of the curve.

1 $$\tau\:=\:\frac{{d}_{curved}}{{d}_{straight}}$$

To obtain the macropores, the original segmentation is inverted, and all macropores are separated by combining watershed segmentation with distance mapping. From the distance maps, maxima regions are determined, which serve as the markers for the watershed. To prevent false separation, the contrast factor of the maximum function has been tuned for the best results. At the surface of the segmented material, pores have been separated. These wrong segmentations have been neglected for the following analysis. Afterwards, the connection points are defined as the intersections of the individual macropores. For both the macropores and the connection points, the obtained results are filtered according to their size to eliminate results below the obtained voxel size and above the achievable sizes by the fabrication process. The final calculations of the obtained results are performed in MATLAB (MATLAB R2024a, The MathWorks, Inc., USA).

### Fluid flow simulations

The segmented image stacks are used to generate a mesh for the following fluid flow simulation using Simpleware™ (V-2024.06, Synopsis Inc., Sunnyvale, USA).

The fluid flow in the different porous structures is simulated in COMSOL^®^ (COMSOL Multiphysics 6.3, COMSOL AB, Stockholm, Sweden). This allows a more detailed simulation of the fluid flow even at a fundamental level, compared to simulation methods included in image analysis software. Here, the stationary, linear water flow through the structure is simulated. For all investigated samples, the Re-numbers are < 1; therefore, a laminar flow simulation is reasonable. As the simulation is simplified, the wetting properties and capillary forces are not considered. The boundary conditions are set to an input pressure of 1.5 atm and an output pressure of 1 atm at the upper and lower interfaces of the pore networks, respectively. For the sample borders in the xy plane, the boundary condition is set as no slip. The initial condition is set as 1 atm.

To correlate the simulated velocities with the connection point diameters, the velocity distribution in the sample is imported into MATLAB along with the locations of the connection points. By assigning velocity locations to different connection points, a correlation between connection point size and simulated velocity is established.

## Results and discussion

**Fig. 3:**
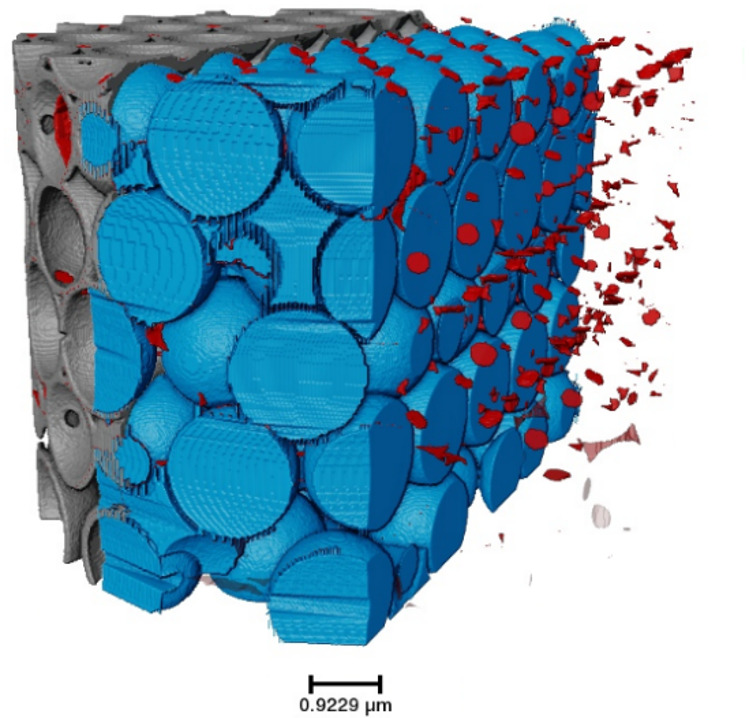
The isoporous 3D structures are differentiated into three components: the material (grey), the macropores (blue), and the connection points (red). A volume rendering of the PXCT data is shown

In general, the analyzed isoporous 3D nanostructures can be characterized by the material, the macropores, and the connection points, as shown in Fig. 3. The solid material is indicated in grey, enclosing the air inside the sample. The macropores (blue) are the large spherical air regions inside the material, while the connection points, shown in red, are the intersections between the different macropores.

### Ordered isoporous 3D structures

**Fig. 4:**
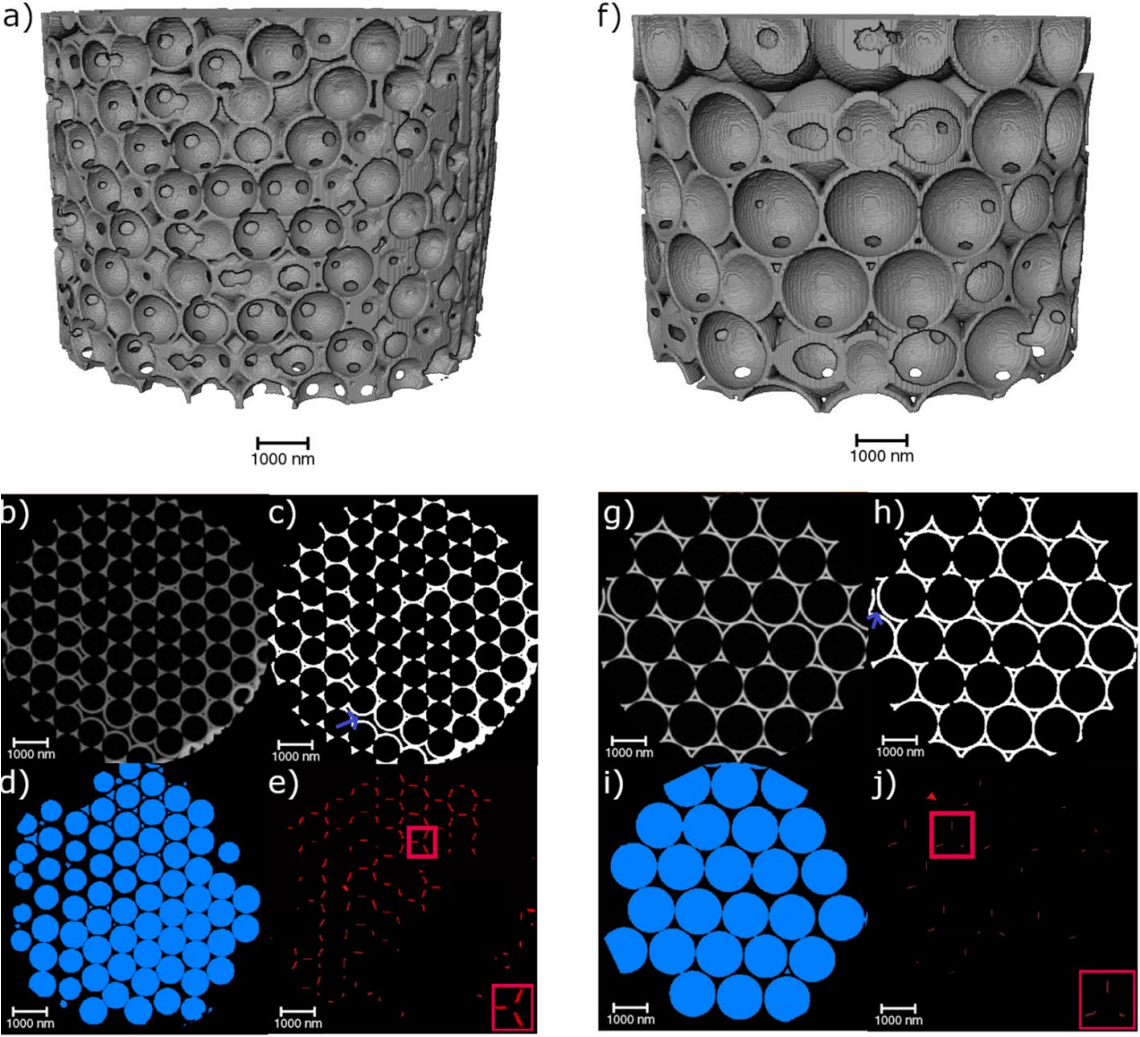
Overview of the image processing and the resulting image stacks. On the left (**a**–**e**), the 900 nm ordered nanostructures are shown, whereas the 1500 nm ordered nanostructures are given on the right (**f**–**j**). A 3D representation of the imaged nanostructures is shown on the top (**a**, **f**). The original, filtered PXCT slices (**b**, **g**) show the achieved stacking with some stacking faults and additional vacancies. From this, the material (**c**, **h**), the macropores (**d**, **i**), and the connection points (**e**, **j**) were segmented and calculated. Exemplary defects are indicated by blue arrows (**c**, **h**)

The ordered isoporous 3D structures manufactured using particles of different diameters are visualized in Fig. 4. As can be seen, both structures are highly ordered. However, they contain small defects, known to originate from the template self-assembly process, such as stacking faults and vacancies [28]. Comparing the obtained segmentations of material, macropores, and connection points, the difference in the size of the connection points becomes apparent. As is expected, the 1.5 μm pore structure’s connection points are significantly lower due to the overall lower number of macropores. However, the size of the connection points in the 1.5 μm pore structures appears smaller.

Table 1 shows the surface area and volume fraction of both ordered macropore samples. For the calculation and analysis of the material, the outer surfaces of all samples are neglected.

**Table 1:** Surface area and volume fraction of the ordered isoporous structures

	900 nm ordered macropores	1500 nm ordered macropores
Surface area / µm²	1604.21	1208.10
Active surface area / µm²	998.49	677.61
Volume fraction material / %	25.78	21.20
Volume fraction air / %	74.22	78.8
Surface area/ volume / 1/µm	3.6	2.7

The surface areas of the samples show that the 900 nm ordered sample has a significantly larger surface area than that of the 1500 nm ordered sample, which was expected. However, it must be kept in mind that all surfaces are considered in this calculation, even though not all surfaces contribute to the open porous structure. Some surfaces belong to interstitial pores without a connection to other pores, as seen in Fig. 4a and f. Therefore, they are of no importance regarding the fluid flow inside the material. Considering only the active surface participating in the fluid flow, a similar trend appears. This means that for the 900 nm ordered structure, 93% of the macropores participate in the fluid flow, while for the 1500 nm ordered structure, it is only 75% of the macropores. Additionally, the volume fraction of the material is lower for the sample with bigger macropores.

**Table 2:** Assumptions and theoretical packing values for the ordered structures

	900 nm ordered macropores	1500 nm ordered macropores
Packing density / %	74	74
Wall thickness / nm	90	80
Volume fraction air / %	63.9	78.7
Surface area/ volume / 1/µm	8.1	5.3

Comparing the obtained fractions and surface area-to-volume ratios to the theoretical values given in Table 2, differences are seen. In the case of the bigger macropores, air has a volume fraction of 78% which fits well with the theoretical value. In the case of the smaller macropores, a higher value is achieved. This may be due to a higher variation in wall thickness than assumed in the theoretical calculations. In both cases, the theoretical surface area/ volume is higher than the actual value. As the theoretical assumption does not consider surface roughness effects, the differences can be attributed to this.

**Fig. 5:**
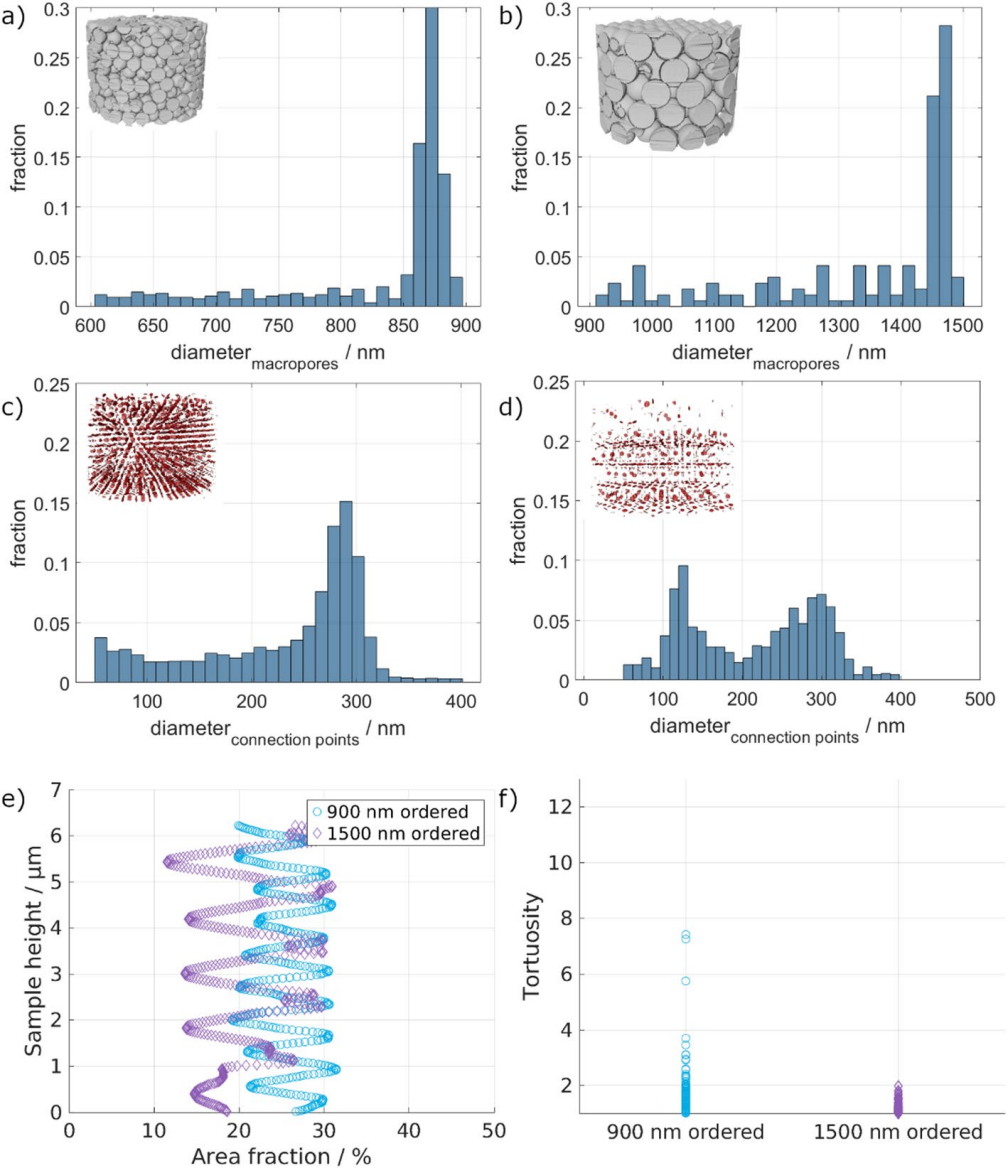
Macropore diameter distribution of the ordered 900 nm (**a**) and 1500 nm (**b**) pore structures. Connection point diameter distribution of the ordered 900 nm (**c**) and 1500 nm (**d**) pore structures. **e** Pore area fraction over the sample height of the ordered structures showing the periodic arrangement of the macropores. f) Tortuosity distribution of the ordered samples through the isoporous network

Next to the surface area and volume fraction, the size distributions of both the macropores and connection points are of importance. The respective histograms are shown in Fig. 5a–d.

The macropore diameter distribution of the 900 nm ordered sample shows that most macropores have 850–900 nm diameters. Similarly, for the 1500 nm ordered sample, most macropores have 1400–1500 nm diameters. Overall, this indicates that the desired diameters of 900 nm and 1500 nm were achieved rather well. Due to the significantly larger pores of the 1500 nm ordered sample, the total number of pores is much lower, as the PXCT samples have an overall similar size. The difference in nominal particle diameter and pore diameter results from the shrinkage following the burnout process during the sample preparation [7]. The shrinkage of the smaller macropore sample is 7.76%, while it is slightly higher for the 1500 nm ordered sample (10.55%), indicating a template size dependence concerning the total shrinkage. Gomez-Gomez et al.. have also reported on the influence of the initial template size, and thus macropore size, on the structural changes upon annealing of similar ALD-based isoporous samples [29].

Unlike the previous size distribution, a different behavior is observed for the diameter distribution of the connection points. For the 900 nm ordered sample, most connection points have a diameter between 250 and 300 nm. However, a significant number of connection points have diameters below 250 nm. Above 300 nm, there are only very few connection points. A different trend is seen for the bigger macropore sample. Here, two different maxima occur for the connection point diameter, at ~ 120 and ~ 300 nm. Neither maxima is as pronounced as the previously seen maximum for the smaller sample. This indicates that the distribution of the bigger macropores is not as defined as that of the smaller macropores. The overall number of connection points is lower for the bigger macropore sample due to the reduced number of macropores, as previously discussed.

Looking at the periodicity of the porous structure (Fig. 5e), no notable inhomogeneities regarding the area fraction at different heights are seen. Except for small irregularities at the bottom of the larger pore structure, the area fraction of the material is homogeneously periodic over the whole sample height.

In general, tortuosity is defined as how twisted the paths inside the structure are. In the present work, the tortuosity through the isoporous structure is defined by the skeleton of the different structures. The tortuosity is lower for the 1500 nm ordered sample than for the 900 nm ordered sample. This indicates that the fluid can flow on shorter pathways through the material. In contrast, the higher number of connection points and macropores in the 900 nm ordered sample leads to a significantly higher tortuosity than the 1500 nm ordered sample.

**Fig. 6:**
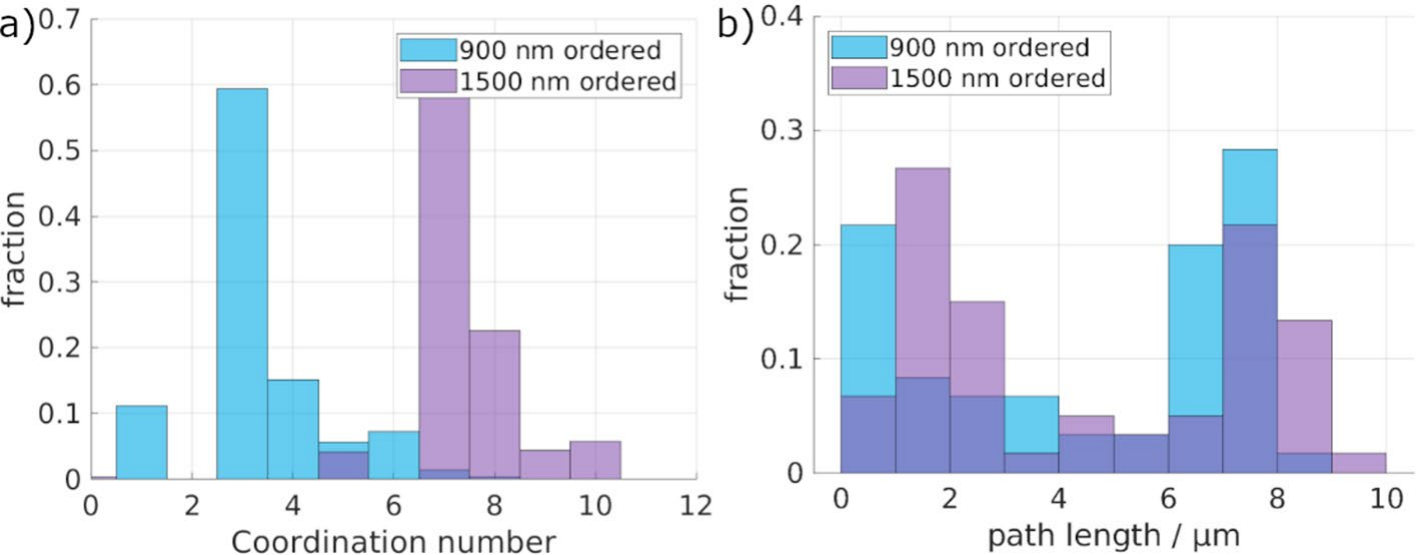
**a** Shown are the coordination number distributions of the ordered macropores. **b** Given are the path length distributions of the ordered macropores, as determined by the fluid simulation

Comparing the coordination numbers (Fig. 6a) of the ordered structures, surprisingly, a higher coordination number for the 1500 nm structure is seen. Additionally, the possible path lengths in the samples show a bimodal distribution in both cases. In the case of the 1500 nm sample, a high number of paths have short path lengths, shorter than the sample height of ~ 6 μm. Therefore, there may be a higher number of possible paths as given by the coordination number. However, not all paths lead through the whole sample, explaining the lower overall tortuosity.

**Fig. 7:**
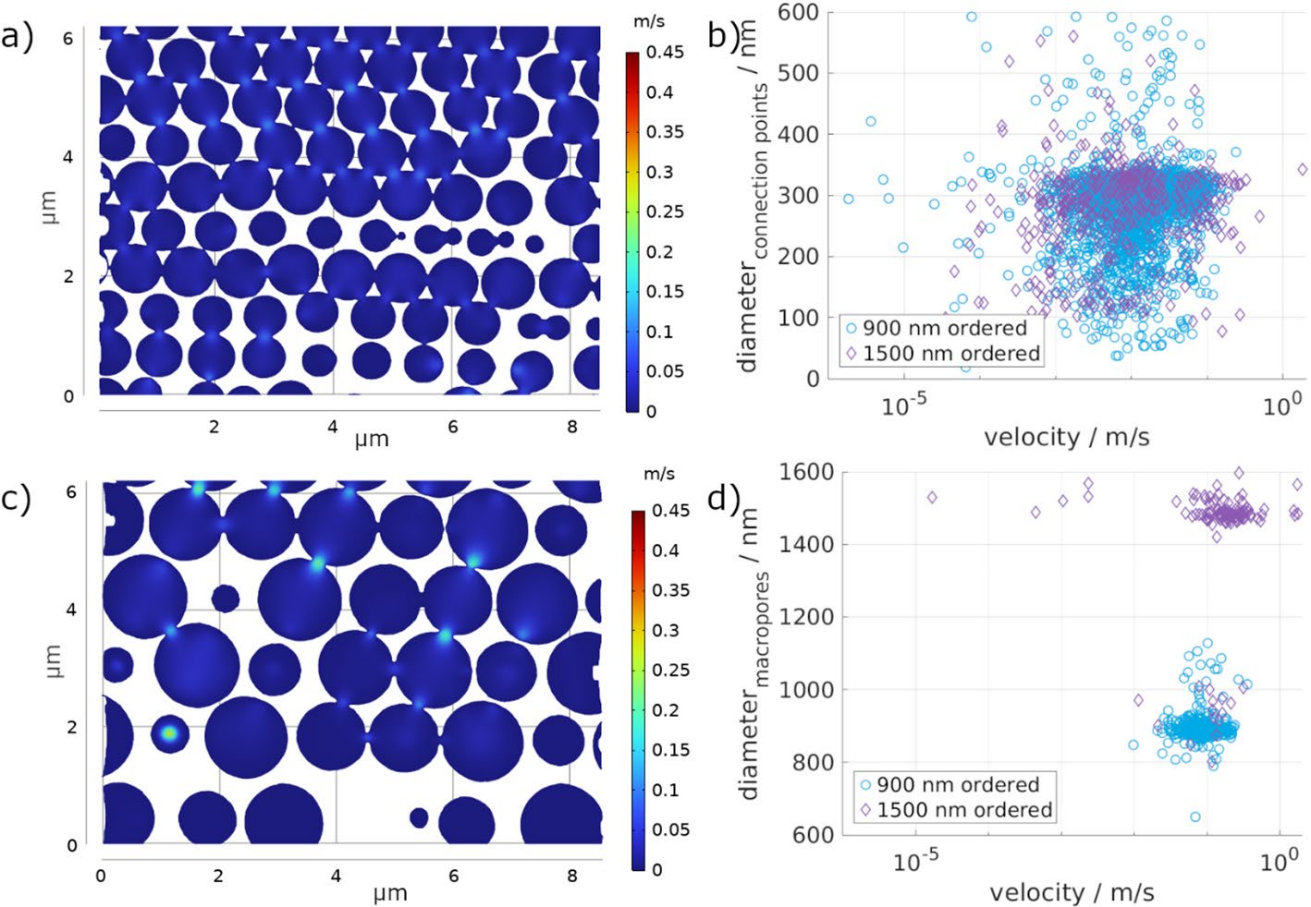
Fluid flow simulation of the 900 nm ordered sample (**a**) and the 1500 nm ordered sample (**c**). Depicted is the fluid velocity in one representative slice in the material, with the fluid flowing from top to bottom. The occurring fluid velocities are correlated to the connection point diameter (**b**) and the macropore diameter (**d**)

To analyze the fluid flow through the material, only the active pores, participating in the fluid flow, were used in the stationary, linear flow simulation. As the voxel size of the original tomograms was 14.53 μm, only structures above the resulting resolution are considered. Nevertheless, structures below the resolution may exist. In Fig. 7, a representative slice through the material showing the resulting velocity distribution is depicted for the ordered samples with 900 nm macropores (a) and 1500 nm macropores (b). The simulation shows that the average fluid velocity is similar for both pore sizes, with 12.64 × 10^− 3^ m/s and 12.11 × 10^− 3^ m/s for 900 nm and 1500 nm, respectively. Similarly, the permeability of the 900 nm (1553 nm²) and the 1500 nm (1488 nm²) ordered samples is similar.

Even though the average fluid velocities and permeability values were similar for both macropore sizes, a slight tendency to higher velocity values with increasing macropore size is observed in Fig. 7d. Moreover, the maximum occurring fluid velocity of the 1500 nm ordered structure (1.91 m/s) is more than double that of the 900 nm ordered structure (0.36 m/s). This indicates that there may be one main pathway through the material for the.

1500 nm ordered sample, which is supported by the low tortuosity observed for this sample (Fig. 5f). As reported by Farahani & Nezhad [30], ordered structures have less tortuosity, therefore leading to fewer obstructions for fluid flow. Moreover, the reduced tortuosity is associated with less drag, i.e., less friction, so the fluid can flow in preferential channels with inertia, leading to faster fluid flow. This observation highlights that in this study, the macropore dimensions influence the fluid transport at the submicron scale more than the connection points’ size (later discussed). This can be associated with smaller macropores increasing the number of connection points for the same sample volume, which is likely the reason for the observed increase in tortuosity. In fact, tortuosity is fundamentally defined as the ratio of the average actual fluid path length to the straight-line length [31]. Therefore, a greater density of connection points, i.e., a greater density of obstacles, shall elongate the length of the path followed by fluid, resulting in higher tortuosity. According to established models for porous media flow [32, 33], increasing tortuosity impacts fluid transport, as highlighted by the Darcy law (Eq. 2) and the Kozeny-Carman equation (Eq. 3) [34].

2 $$u=-\frac{\kappa\:}{\mu\:}\nabla\:\mathrm{p}$$

3 $$\kappa\:=\frac{{d}_{p}^{2}}{180}\frac{{\epsilon\:}_{p}^{3}}{{(1-{\epsilon\:}_{p})}^{2}}$$

Here, *u* is the Darcy’s velocity, $\kappa\:$ the permeability, *µ* the fluid’s dynamic viscosity, *p* the pore pressure, *d*_*p*_ the particle diameter, and ${\epsilon\:}_{p}$ the porosity. On the one hand, Darcy’s law shows a linear proportionality between the volumetric flow rate and the permeability. On the other hand, the Kozeny-Carman equation expresses permeability as inversely proportional to the square of tortuosity. Consequently, a greater tortuosity translates into a reduced permeability, which reduces the volumetric flow and thus the maximum achievable velocity in porous structures. However, permeability values remain similar, since the permeability simulation is averaged over the whole structure, i.e., our simulation does not allow for assessment of localized permeability values. This suggests that in this work, permeability is primarily influenced by average structural characteristics, such as overall connectivity and connection points distribution, rather than local geometric variations.

The relationship between fluid velocity and connection point sizes (Fig. 7b) directly correlates with the connection points’ size distributions (Fig. 5c and d). In Fig. 7b, it is possible to see that both for the 900 nm and the 1500 nm ordered porous structure, there is a clustering of the simulated velocities around the peak values of the connection points’ size distribution. This is clearer for the connection point size of 300 nm (for both) and less pronounced at 120 nm for the 1500 nm sample. Nevertheless, it is clearly seen that for both samples a broad distribution of simulated velocity values is present, regardless of the connection point size, i.e., there is no clear trend between the increase of connection point size and velocities. Exemplarily, taking the simulated velocity of 0.01 m/s, connection point sizes from about 50 nm up to 600 nm can be found.

Looking at the correlation of the velocities with the connection point diameters (Fig. 7b) and the macropore diameters (Fig. 7d), a broad distribution of velocities and connection point sizes occurs for both samples. In contrast, only a small range of macropore sizes participates in the fluid flow with a lower variation in velocities. Nonetheless, the simulated velocities for the 1500 nm ordered structure have a broader range, as seen in Fig. 7d, confirming the assumption of one more direct pathway with higher velocities and several pathways with higher tortuosity. Due to the large size of the macropores, only a few stacking layers are present for the 1500 nm ordered structure compared to the 900 nm ordered sample. These lead to a smaller number of connections, and in combination with the ordering that was achieved, the pathways can be short and directly aligned. In contrast, there are significantly more possible pathways in the 900 nm sample, leading to a more homogeneous velocity distribution with fewer extrema.

Disordered isoporous 3D structures.

**Fig. 8:**
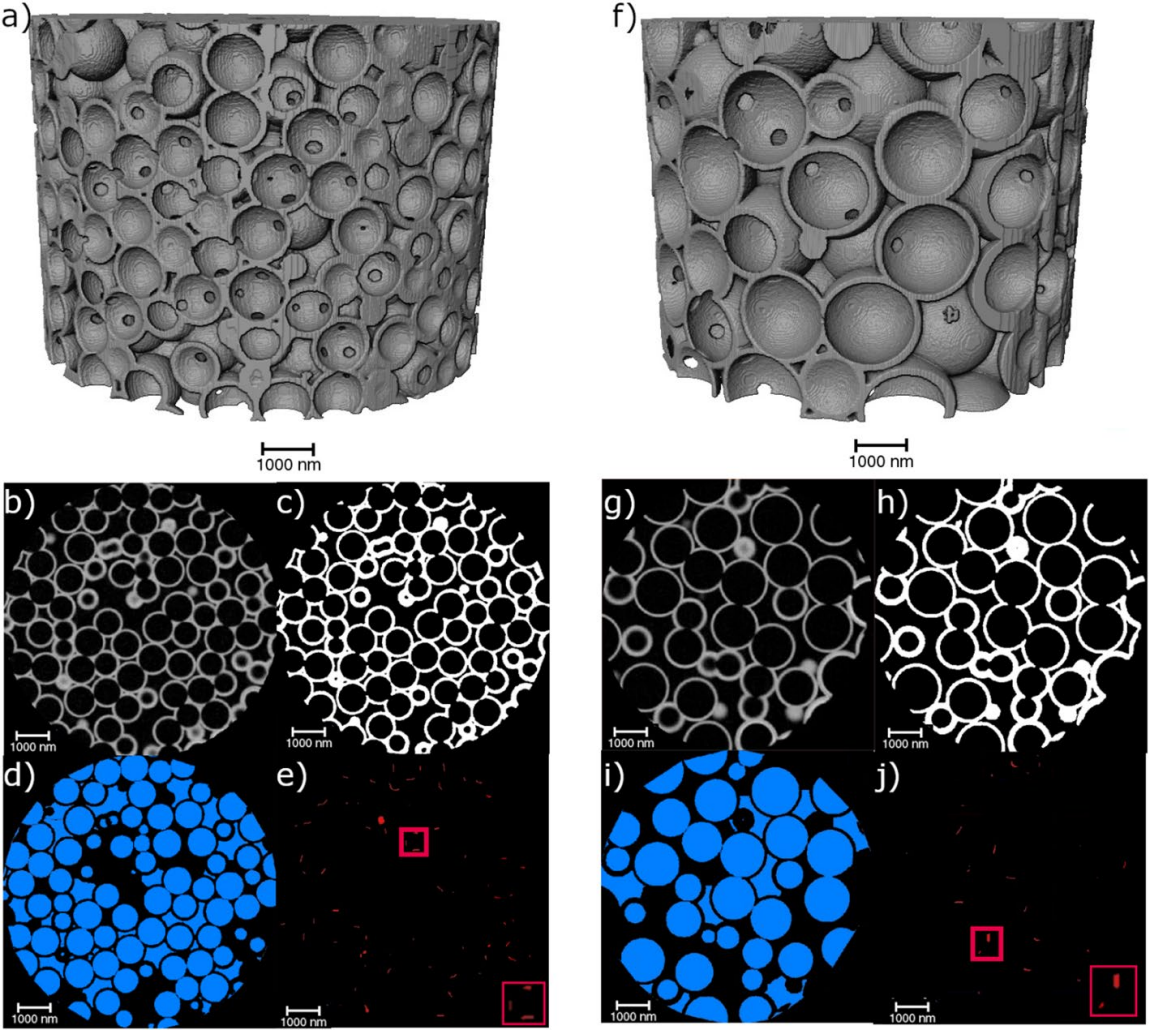
Overview of the image processing and the resulting image stacks. On the left (**a**–**e**), the 900 nm disordered samples are shown, whereas the 1500 nm disordered structures are given on the right (**f**–**j**). A 3D representation of the imaged nanostructures is shown on the top (**a**, **f**). The original, filtered PXCT slices (**b**, **g**) show the achieved stacking with some stacking faults and additional vacancies. From this, the material (**c**, **h**), the macropores (**d**, **i**), and the connection points (**e**, **j**) were segmented and calculated

A similar analysis was performed for the disordered structures. Exemplarily, tomographic slices with the individual segmentation results are given in Fig. 8. Unlike the ordered nanostructures, large interstitial pores are present due to the disordering, which was expected. As shown in previous studies, these interstitial pores can be differentiated into big and small voids. The big voids are mainly seen in the present nanostructures and can be attributed to the self-assembly defects formed during drop casting [5, 35].

Similar to the ordered isoporous structures, the surface area and active surface area of the 900 nm disordered sample are significantly larger than those of the 1500 nm disordered sample. Additionally, the volume fraction of the material is larger for the 900 nm disordered sample than for the 1500 nm disordered sample. Looking at the number of macropores participating in the fluid flow, the difference between 900 nm disordered (75%) and 1500 nm disordered (70%) is small (Table 3).

**Table 3:** Surface area and volume fraction of the disordered isoporous structures

	900 nm disordered macropores	1500 nm disordered macropores
Surface Area / µm²	1787.99	1138.89
Active Surface Area / µm²	1095.59	555.82
Volume Fraction material / %	28.53	22.25
Volume Fraction air / %	71.47	77.75
Surface area/ volume / 1/µm	3.87	2.43

As before, the volume fraction of air of the 1500 nm disordered sample fits well with the theoretical value when a packing density of 64% is assumed. The air volume fraction of the 900 nm disordered sample is smaller than the theoretical value, which, again, can be attributed to the variations in the wall thickness. This also applies to the lower surface area/ volume ratios, where surface roughness is neglected in the theoretical calculation (Table 4).

**Table 4:** Assumptions and theoretical packing values for the disordered structures

	900 nm disordered macropores	1500 nm disordered macropores
Packing density / %	64	64
Wall thickness / nm	90	100
Volume fraction air / %	68.8	77.7
Surface area/ volume / 1/µm	7.0	4.5

**Fig. 9:**
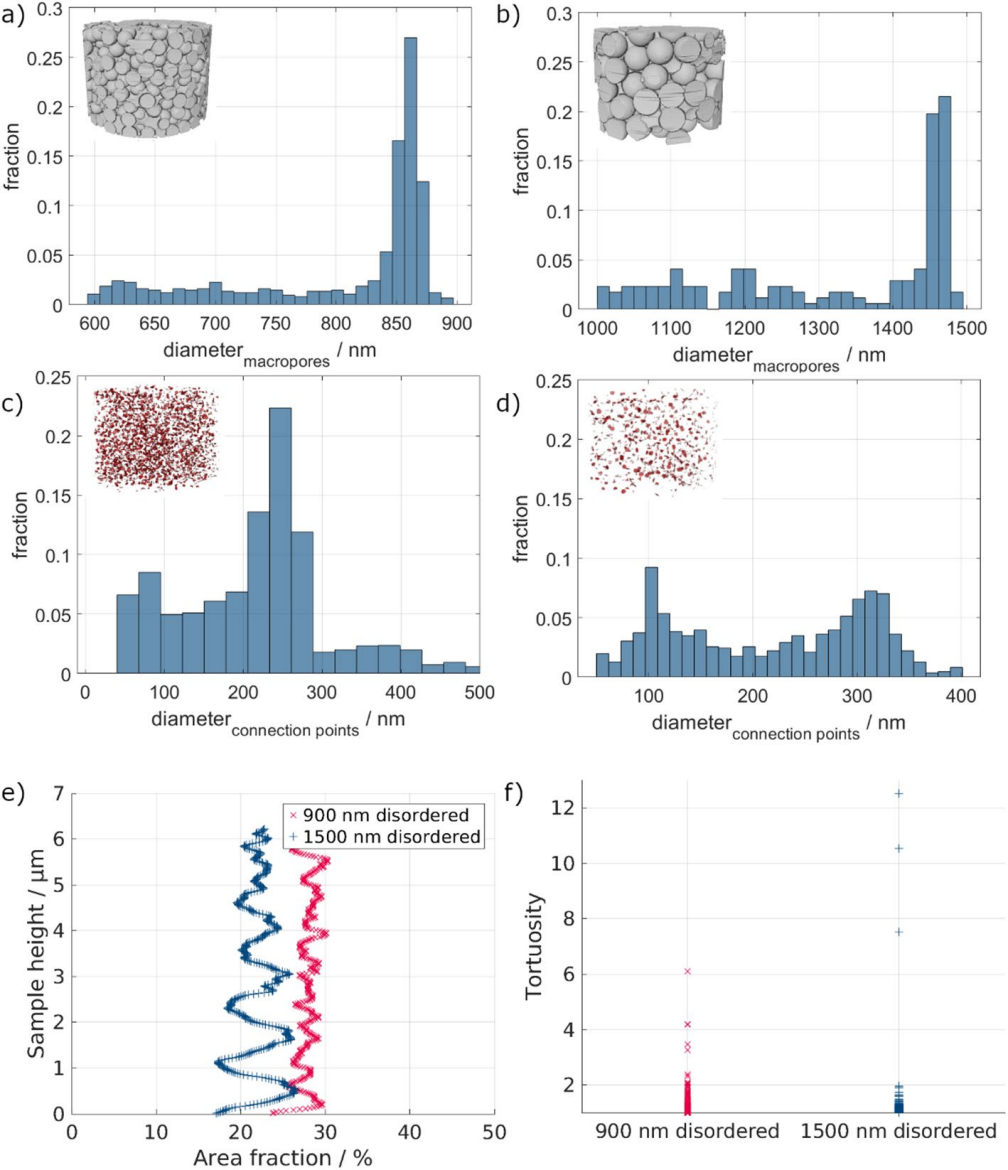
Macropore diameter distribution of the disordered 900 nm (**a**) and 1500 nm (**b**) structures. Connection point diameter distribution of the disordered 900 nm (**c**) and 1500 nm (**d**) pore structures. **e** Pore area fraction over the sample height of the disordered structures showing the periodic arrangement of the macropores. **f** Tortuosity of the disordered samples through the isoporous network

The macropore diameter distribution of the smaller disordered structure (Fig. 9a) shows that most macropores have a diameter of 830–900 nm, with only a small number of macropores having smaller diameters. In the case of the bigger disordered sample (Fig. 9b), most of the macropores have a diameter between 1400 nm and 1500 nm. As for the 900 nm disordered sample, the amount of smaller-sized macropores is low. The average shrinkage of the 900 nm disordered sample is 10.53%, and 11.95% of the 1500 nm disordered sample. For the connection points of the 900 nm disordered sample, the distribution is broader than for the macropore diameter. The majority of connection points have a diameter of 200–300 nm, whereas there are a high number of connection points with a diameter between 50 nm and 200 nm. Only a small number of connection points have a diameter above 300 nm. Unlike the 900 nm disordered sample, the 1500 nm disordered sample does not have a clear maximum in the histogram (Fig. 9d) regarding the diameter of the connection points. Here, two slight maxima around 120 nm and 320 nm are detected. However, a smaller number of connection points have diameters between these maxima. Comparing the results of both the ordered and disordered samples, only marginal differences between the ordering states are seen with respect to the connection point and macropore sizes.

This behavior is related to the previously mentioned template self-assembly defects. As previously reported, the self-assembly of colloids by vertical convective self-assembly is more challenging in the case of larger particles due to higher sedimentation velocity [36].

The periodicity of the structures is much more inhomogeneous, which indicates the disordered character of the 3D isoporous structure. Additionally, a vertical variation is detected for the larger pore structure. At the bottom of the sample, a higher area fraction of the material is present. This means that a separation between larger and smaller macropores occurred during the fabrication, meaning that the macropore sizes are not homogeneously distributed over the height of the sample.

**Fig. 10:**
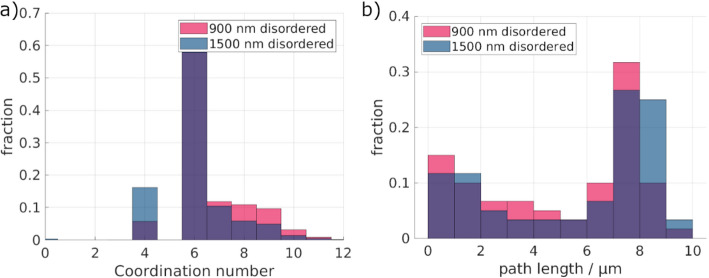
**a** Shown are the coordination number distributions of the disordered macropores. **b** Given are the path length distributions of the disordered macropores, as determined by the fluid simulation

Unlike the coordination number distribution of the ordered structures, no significant differences between the disordered 900 nm and 1500 nm structures are seen in Fig. 10a. For the path lengths (Fig. 10b), slightly longer paths are detected compared to the ordered structures. Nevertheless, the paths are overall longer, correlating well with the overall higher tortuosity.

**Fig. 11:**
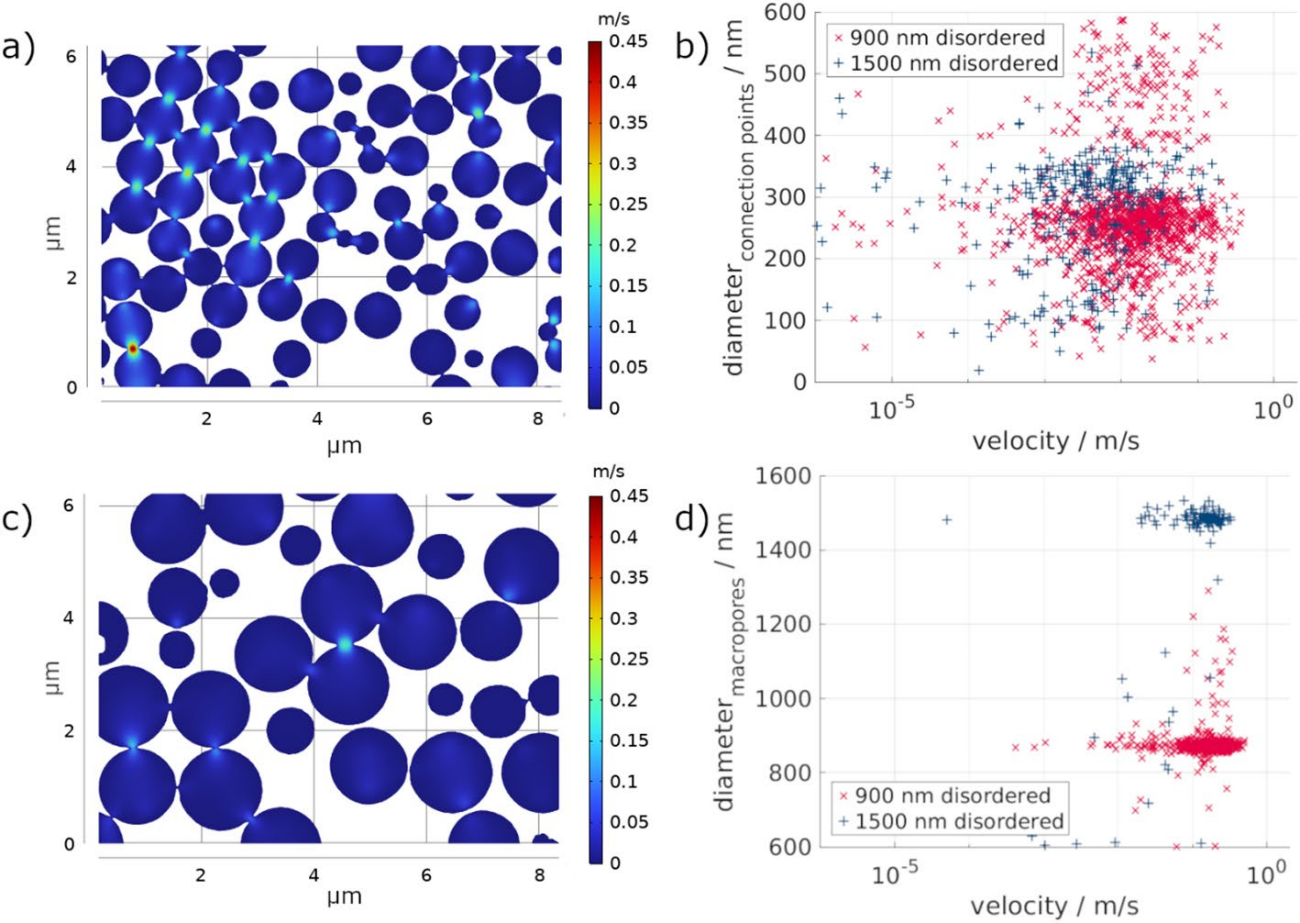
Fluid flow simulation of the 900 nm (**a**) and the 1500 nm (**c**) disordered sample. Depicted is the fluid velocity in one representative slice in the material, with the fluid flowing from top to bottom. The occurring fluid velocities are correlated to the connection point diameters (**b**) and the macropore diameters (**d**)

Figure 11 shows one representative slice of the fluid flow simulation in the disordered (a and c). As expected, the macropores are less homogeneously distributed, leading to less interconnection between the individual pores. This is particularly pronounced for the 1500 nm disordered sample, where the average velocity (7.71 × 10^− 3^ m/s) is significantly lower than the 900 nm disordered sample (17.87 × 10^− 3^ m/s). The maximum velocity is also lower for the 1500 nm disordered sample (0.32 m/s) than the 900 nm disordered sample (0.51 m/s). Combining the disordering of the macropores with the pore size leads to the opposite effect as for the ordered samples. The 1500 nm disordered sample has fewer connections between the macropores, with a high tortuosity (Fig. 9f), due to fewer and less direct pathways, thus leading to slower fluid velocities. The correlation between tortuosity and permeability has also been observed by other authors [32, 33]. In agreement with these previous works, we observe a low permeability (947 nm²) and lower average fluid flow velocities. We associate this with increased drag on the fluid, as previously reported [30]. In contrast, the smaller 900 nm disordered sample has the highest permeability of all investigated samples (2196 nm²) with the highest average fluid flow velocity. This is related to the alignment of the pores and their connection points, which lead to lower tortuosity in this specific sample. In some regions of the sample, the 900 nm pores are aligned nearly vertically below each other, enabling the direct flow from one pore into the other (see top left in Fig. 11a), forming preferential channels for fluid flow, with likely less drag, leading to faster fluid flow velocity.

The correlation between the simulated velocities and the connection point and macropore sizes (Fig. 11b and d) shows that, again, the distribution of connection point sizes and occurring velocities is broad for both disordered samples. Similarly to the ordered samples, a cluster of simulated velocities matches the peak of the connection points’ size distribution, especially for values around 300 nm of connection points’ size. As for the ordered 900 nm structure, the disordered one shows an increase in the frequency of velocities with increasing connection points’ diameter, attesting once more to the clustering. However, the macropore sizes are very limited, with only a small velocity variation. The occurring velocities are even more equal for the 1500 nm disordered sample.

In contrast to the surface area and volume observations, where only small variations between the ordered and disordered structure properties were seen, larger differences are present in the correlation. The correlation between the structural parameters and the fluid flow simulations demonstrates that the connection point size is the most important. Due to their small sizes, the connection points determine the average fluid velocity in the material. Small connection points lead to an increase in the velocity, while larger connection points reduce the fluid velocity, as was also shown before [37]. In addition, the number of connection points and their average diameter distribution play a major role in the fluid flow. Samples with higher numbers of connection points led to higher permeabilities and average velocities in the material. In fact, both 900 nm porous structures turn out to be the most permeable structures, which differs from what was observed by Pham et al. for metallic isoporous structures, where greater pore diameters lead to greater fluid transport properties [8]. This difference can be explained by the role of the connection points’ size distribution. As mentioned, the 900 nm porous structures were characterized by a monomodal diameter distribution centered around 300 nm. In contrast, the 1500 nm structures were characterized by a bimodal distribution with two maxima, a similar one around 300 nm and another at smaller diameters around 120 nm. We hypothesize that these smaller connection points’ diameters create additional drag, slowing down the fluid flow in the structure, leading to both 900 nm samples being more permeable than the 1500 nm ones. Most importantly, it indicates that permeability in the ceramic isoporous structure hereby studied is more strongly influenced by the presence and frequency of connection points rather than the macropore size alone. This effect aligns well with other studies, where the relevance of the connection point sizes’ influence on the fluid flow behavior was studied [13, 14]. Nevertheless, as was mentioned before, important influencing factors such as wetting and capillary forces may further affect the simulated fluid flow. This material-fluid interaction was neglected in the presented study and will be employed in future works to enhance the understanding of the presented correlations.

The disordered 900 nm structure also has larger permeability than its ordered counterpart. This is related to the observed macropores’ alignment leading to locally less tortuous paths, as explained above, thus reducing local fluid flow resistance. A high tortuosity leads to low maximum velocities in the material, as seen for the 1500 nm disordered sample. Since not all macropores were interconnected due to the disorder, the tortuosity increased, and the average and maximum velocity decreased. In contrast, the 1500 nm ordered sample had a low tortuosity due to the limited possibility of assembling large pores in an ordered way and fewer possible pathways. The size of the macropores has only a minor influence on the fluid flow. As was shown, the 1500 nm ordered and disordered samples exhibit opposing effects, indicating that the ordering primarily determines the fluid properties of the material rather than the macropore size. This tendency is supported by previous observations showing that the ratio between connection point and macropore size plays a role in the flow inside the macropores [12]. By increasing the connection point size, particles can more easily pass through the pores, improving the fluid flow. This can also be detected in the present study, as the smaller macropore samples have a smaller mismatch between connection point size and macropore size. Due to this, the velocity inside the macropores is higher, and a narrower velocity distribution is achieved. In future experiments, we aim at systematically investigating the influence of the connection points’ diameter, also known as pore throats or throat diameters, by altering the pore throat diameter by heat treatments. This can be performed in the templates prior to ALD, leading to enlargement of the particle’s necks, as first demonstrated by Gates et al. [38] or in the porous structures after template removal, as previously observed and described in our own work [7]. In both cases, it is expected that the macropore diameter is also altered, and even though the alteration of the macropores’ diameter in relative % is smaller than the relative % alteration of the pore throat diameter, this should be taken into account when interpreting the results.

Overall, the obtained results show only the behavior of the fluid flow at a microscale, whereas typical applications use samples of much larger dimensions. Therefore, the results have to be related to the macroscopic scale. Nevertheless, the macroscopic pore structure is applicable to the present samples, offering initial possibilities to tune the porous structures. Additionally, some applications rely on porous structures with mesoscale pores. These pores are much smaller than the investigated samples. At these length scales, the fluid flow is highly dominated by surface-fluid interactions, as was stated before. Therefore, a more advanced simulation including these interactions is needed to analyze the behavior at smaller scales. Nevertheless, the influencing factors of the geometry can be related to the mesoscale structures.

## Conclusion

Using synchrotron radiation-based PXCT, ordered and disordered 3D isoporous structures with 900 nm and 1500 nm pore sizes were characterized. It was shown that the applied processing routes led to homogeneously sized pores with roughly the desired template pore size. Additionally, it was possible to characterize the connection points. The size distribution of both the macropores and connection points showed no significant differences between the ordered and disordered samples of the same pore size. However, the obtained 3D images from the PXCT were used for a fluid flow simulation of water through the different isoporous structures. Here, significant differences in fluid flow behavior were detected. The characterization regarding their permeability, tortuosity, and fluid velocity showed that the connection point size, in combination with the ordering state, is the most important parameter. As was known before, the connection point size is the most important parameter to tune the fluid flow in porous structures. It was shown that this is even more important than the ordering of the macropores. In contrast, the macropore size plays only a secondary role. Indeed, the samples with similar pore size but opposing ordering also showed opposing fluid flow behavior. To better understand the underlying process, further experiments are needed to confirm these results and gain deeper insights into the fluid flow in these structures. It must be noted that the applied PXCT technique is reasonable in the current study, as a homogeneous structure is investigated. For less homogeneous structures, the necessary small sample sizes might not be sufficient for a complete representation of the structure. In the future, a fluid flow simulation considering important parameters like wetting and capillary forces will be implemented to confirm the preliminary results. Additionally, the simulations will be further validated by experimental verifications. These findings can help in the future design and tailoring of 3D isoporous structures for specific applications.

## Supplementary Information

Below is the link to the electronic supplementary material.

Supplementary Material 1

## Data Availability

The data that support the findings of this study are available from the corresponding author upon reasonable request.
