# Supplementary material for: High-resolution analysis of ordered and disordered isoporous 3D nanostructures using PXCT
Source: Discov Nano. 2026 Feb 17;21(1):46. doi: 10.1186/s11671-026-04435-7 (PMC12913824; doi:10.1186/s11671-026-04435-7)
Supplement: Supplementary file 1 — Supplementary Material 1 [file 11671_2026_4435_MOESM1_ESM.docx]

# Supplementary Information

### Ptychographic X-ray computed tomography (PXCT) data acquisition and processing

Measurements were performed at the cSAXS beamline of the Swiss Light Source at the Paul Scherrer Institute in Villigen, Switzerland, at a photon energy of 6.2 keV. This energy was chosen because it provides the highest coherent flux at the cSAXS beamline. Moreover, at this energy, the transmission through the sample was higher than 40%, which guarantees a dose-efficient measurement.

We used an instrument specifically designed for high-resolution PXCT [1], which we call flOMNI. The samples were measured at room temperature with a constant flow of N_2_ gas to prevent any possible interaction of the sample material, Al_2_O_3_, with the X-ray beam in the presence of O_2_. For the nitrogen flow, we used the environmental control available in our instrument [2]. Using this environment, one can have the sample in a gas flow and eventually heat the sample up to a temperature of 850 °C while accomplishing PCXT at a spatial resolution better than 20 nm in 3D. In the current work, we used this capability to provide a constant, gentle nitrogen flow to the sample at room temperature. The temperature stability was determined by the typical temperature control in the hutch, which is about +/- 0.3 °C. The illumination on the sample was produced by a coherently-illuminated Fresnel zone plate (FZP) of 200 µm diameter and 60 nm outer-most zone width with an integrated central stop of about 50 µm diameter, used in combination with an order sorting aperture of 30 µm diameter, which had a focal distance of 60.0 mm at the used photon energy. The sample was placed about 1 mm upstream of the focal spot of the FZP, where the illumination was about 3.3 µm diameter, with a flux of 1.7×10^8^ photons/s. The FZP had locally misplaced zones, intentionally designed to produce a structured illumination to improve the convergence of ptychographic reconstructions [3]. For ptychography acquisitions, the sample was scanned at several positions with respect to the illumination. For this purpose, a combination of sample and FZP scanning was used, in such a way that the relative position between the sample and the illumination was kept constant during acquisition, while keeping the moving time between acquisitions as short as possible, as described in Ref. [4]. The scan positions followed the pattern of a Fermat spiral [5] with an average step size of about 0.6 µm and with a field of view of 15 µm × 10 µm (horizontal × vertical). At each scanning point, we acquired a diffraction pattern with an Eiger 1.5M detector with a pixel size of 75 µm fabricated by the detector group at the Paul Scherrer Institute [6], which was placed inside a flight tube in vacuum 5.232 m downstream the sample. The exposure time of each diffraction pattern was 0.05 s. In Fig 2, we show an example of a diffraction pattern acquired during a ptychographic scan. For tomographic acquisition of each sample, ptychographic scans were repeated at different rotation angular positions of the sample in equally spaced angular steps. We acquired 600 angular projections for each sample, except for 900 nm ordered, for which we acquired 800 projections. We estimate that a dose of about 1.1×10^8^ Gy was deposited on each sample during data collection, except for 900 nm ordered, for which the dose was about 1.4×10^8^ Gy. The dose was estimated by counting the photons absorbed by the sample, which can be easily done with the same detector used to record the diffraction patterns during tomographic acquisitions, and by measuring the sample mass from the quantitative 3D electron density distributions obtained by ptychographic tomography.

The acquisition parameters for ptychography scans, namely the exposure time and the average step size, were chosen to provide sufficiently high resolution in 2D while keeping the total acquisition time of the tomogram within the measurement time available. We found that an average step size of 0.6 µm and exposure time of 0.05 s provided projections with a 2D resolution of around 28 nm in about 29 s, which was found to be a good compromise. According to the Crowther criterion, one would then need around 600 projections to obtain a similar 3D resolution. For at least one sample, we chose a number of projections that exceeded this number slightly (800), considering that samples were very stable during acquisitions, and there was sufficient time available. Acquiring more projections than strictly needed has the benefit of an increase in signal-to-noise ratio in the final tomogram.


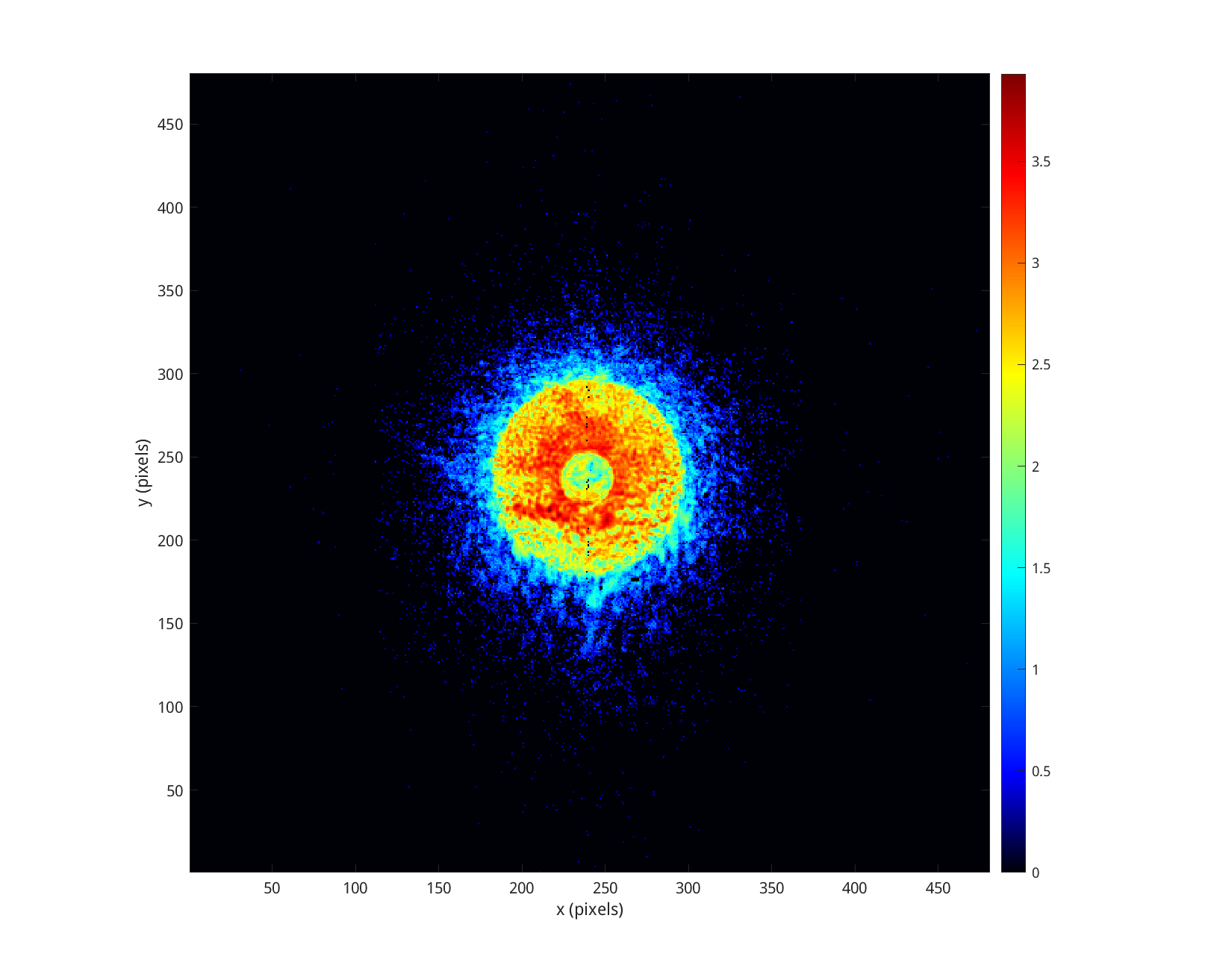


Fig 1: Diffraction pattern acquired during a ptychographic scan at one scan position with an acquisition time of 0.05 s. The color bar shows the number of photons in a log10 scale. The x and y axes are shown in pixels after binning 2x2, i.e. each pixel in this plot corresponds to twice the pixel detector size.


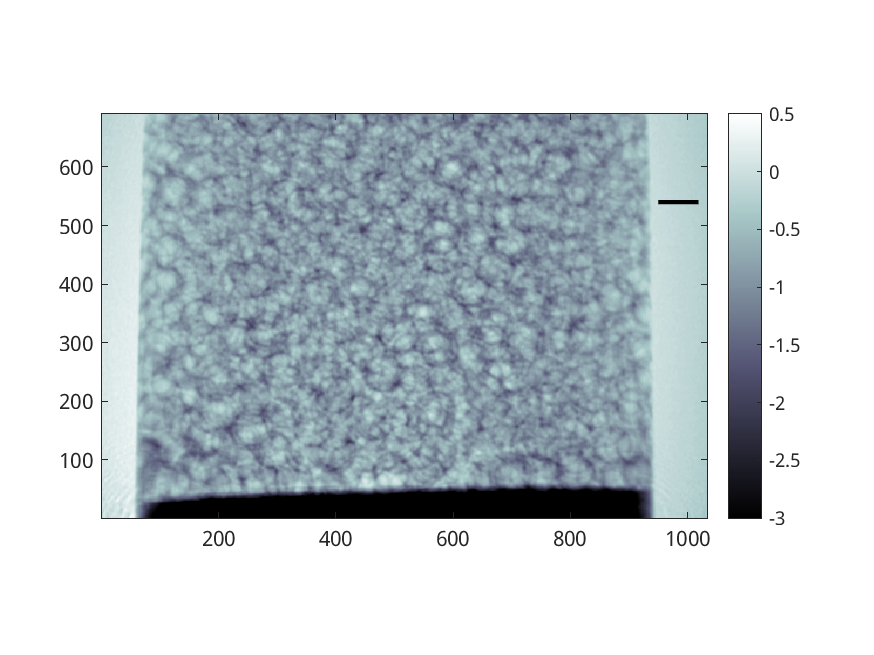


Fig 2: Reconstructed phase projection for one of the samples with a diameter of about
12 µm. The axes indicate reconstructed pixels, with the reconstructed pixel size 14.53 nm. The scale bar is 1 µm, and the color bar indicates phase shift in radians.

The diffraction patterns were cropped to a size of 960×960 pixels around the center and binned in 2×2 pixels for the ptychographic reconstructions. These were performed using 200 iterations of an iterative least-squares solver [7] implemented in the *PtychoShelves* package developed by the Coherent X-ray Scattering group at the Paul Scherrer Institute [8]. The reconstructed images had a pixel size of 14.53 nm. In Fig. 2, we show an example of a reconstructed phase projection. The phase images for each sample were aligned to each other with subpixel accuracy before tomographic reconstruction [9]. In brief, 2D phase projections are first corrected for an offset and a ramp term, which are inherent degrees of freedom in ptychography-reconstructed phase images. After a rough alignment by 2D correlation, a fine vertical alignment is performed using the projected phase along the horizontal direction for all projections. Finally, tomography-consistency is used for the fine alignment of the projections along the horizontal direction. For tomographic reconstruction, we first used filter back projection with a ramp filter, which provided a good initial reconstruction, followed by 10 iterations of the simultaneous algebraic reconstruction technique (SART). The SART refinement provided 3D tomograms with less noise. The 3D isotropic resolution of each dataset was estimated by comparing the Fourier shell correlation between two subtomograms, each computed with half the number of projections, with the half-bit threshold [10]. The resolution of the different datasets ranged from 25 nm to 39 nm. We have additionally checked that line profiles through sharp features of the sample in the 3D datasets are consistent with the resolution estimated by Fourier shell correlation.


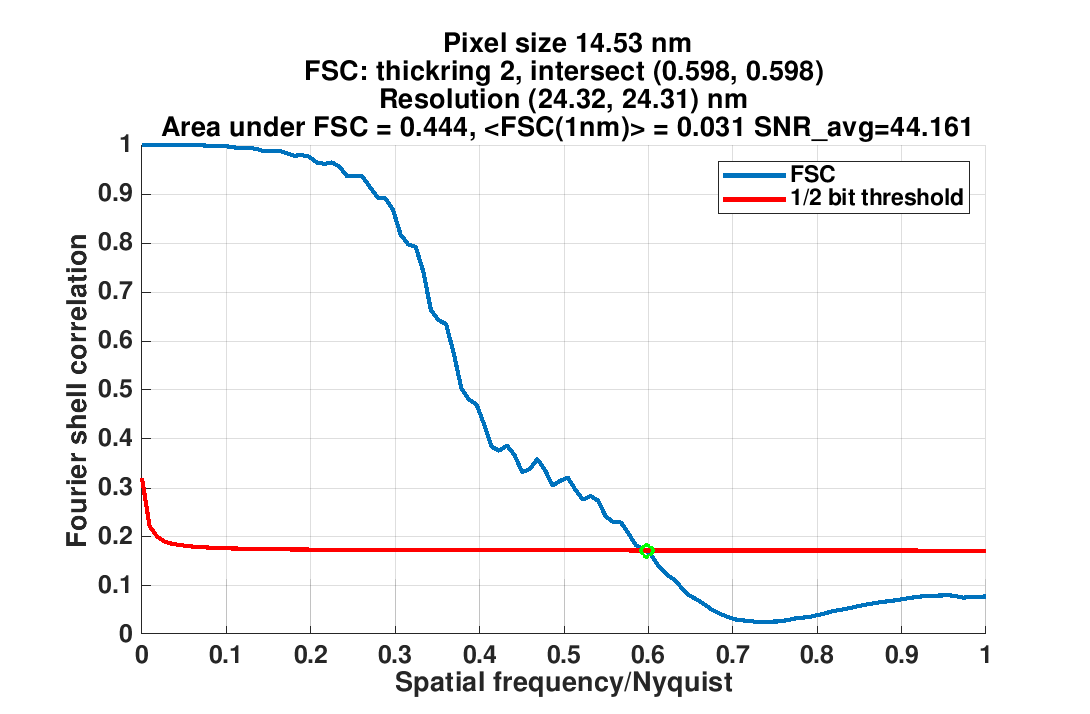


Fig 3: estimation of the 3D resolution of one of the tomographic datasets by Fourier shell correlation (FSC). The blue curve shows the FSC and the red curve shows the ½ bit threshold curve, as described in Ref. [9]. Both curves are plotted as a function of the Nyquist frequency. The point at which both plots cross can be used as an estimation of the 3D resolution, in this example 24 nm.

Fig 4: SEM cross-section imaging of the samples after the template fabrication step by (a,c) vertical convective self-assembly and (b,d) drop casting to generate (a,c) ordered and (b,d) disordered 3D structures, with two different template average particle sizes of (a,b) 0.9 µm and (c,d) 1.5 µm. Scale bars correspond to 10 µm. Note: these are non-post-processed fractured surfaces, thus, different fracture fresh cut planes are seen.

Fig 5: Secondary-electron images taken within the dual-beam FIB device during preparation of the pillars from samples (a-c) ordered 1.5 µm and (d-f) disordered 1.5 µm showing (a,d) isometric view with protective coating deposited, (b,e) exposed pillars from the sample “bulk” and (c,f) extracted pillars after final polishing. Scale bars correspond to 10 µm.

# References:

[1] Holler, Mirko, et al. "Environmental control for X-ray nanotomography." J. Synchrotron Rad. 29, 1223-1231 (2022). https://doi.org/10.1107/S1600577522006968

[2] Holler, M., Diaz, A., Guizar-Sicairos, M. *et al.* X-ray ptychographic computed tomography at 16 nm isotropic 3D resolution. *Sci Rep* **4**, 3857 (2014). <https://doi.org/10.1038/srep03857>

[3] Odstrčil, Michal, et al. "Towards optimized illumination for high-resolution ptychography." *Optics express* **27** 14981-14997 (2019). <https://doi.org/10.1364/OE.27.014981>

[4] Odstrcil, Michal, et al. "Fast positioning for X-ray scanning microscopy by a combined motion of sample and beam-defining optics." *Journal of* *Synchrotron Radiation* **26**, 504-509 (2019). <https://doi.org/10.1107/S160057751801785X>

[5] Huang, Xiaojing, et al. "Optimization of overlap uniformness for ptychography." *Optics express* **22**, 12634-12644 (2014). <https://doi.org/10.1364/OE.22.012634>

[6] Johnson, I., et al. "Eiger: a single-photon counting x-ray detector." *Journal of Instrumentation* **9**, C05032 (2014). <https://doi.org/10.1088/1748-0221/9/05/C05032>

[7] Odstrčil, Michal, Andreas Menzel, and Manuel Guizar-Sicairos. "Iterative least-squares solver for generalized maximum-likelihood ptychography." *Optics express* **26**, 3108-3123 (2018). <https://doi.org/10.1364/OE.26.003108>

[8] Wakonig, Klaus, et al. "PtychoShelves, a versatile high-level framework for high-performance analysis of ptychographic data." *Applied Crystallography* **53**, 574-586 (2020). <https://doi.org/10.1107/S1600576720001776>

[9] Odstrčil, Michal, et al. "Alignment methods for nanotomography with deep subpixel accuracy." *Optics Express* **27**, 36637-36652 (2019). <https://doi.org/10.1364/OE.27.036637>

[10] Van Heel, Marin, and Michael Schatz. "Fourier shell correlation threshold criteria." *Journal of structural biology* 151, 250-262 (2005). <https://doi.org/10.1016/j.jsb.2005.05.009>
